# Nitrogen–sulfur dual-doped porous carbon spheres/sulfur composites for high-performance lithium–sulfur batteries

**DOI:** 10.1039/c9ra00768g

**Published:** 2019-05-28

**Authors:** Liping Zhao, Gang Liu, Peng Zhang, Liqun Sun, Lina Cong, Tong Wu, Bohao Zhang, Wei Lu, Haiming Xie, Hongyu Wang

**Affiliations:** National & Local United Engineering Laboratory for Power Battery, Northeast Normal University Changchun 130024 PR China; Institute of Chemical and Industrial Bioengineering, Jilin Engineering Normal University Changchun 130052 PR China; State Key Laboratory of Electroanalytical Chemistry, Changchun Institute of Applied Chemistry, Chinese Academy of Sciences 5625 Renmin Street Changchun 130022 PR China

## Abstract

A nitrogen–sulfur dual-doped porous carbon spheres/sulfur composite (PCS-NS/S) sample was prepared by a simple hydrothermal method with starch and l-methionine as carbon and nitrogen–sulfur resources, respectively. XRD, XPS, and N_2_ adsorption–desorption tests were used to characterize the crystal and pore structure of the PCS-NS/S sample. The morphology and weight ratio of sulfur were investigated by SEM, TEM, and TG analyses. The sample was used as the positive electrode for lithium–sulfur batteries and found to exhibit excellent electrochemical performance.

## Abstract 2

Simultaneously introduced nitrogen–sulfur through one reagent. The as-prepared PCS-NS/S composites exhibited excellent electrochemical performance as positive electrode for Li–S battery.

## Graphical abstract

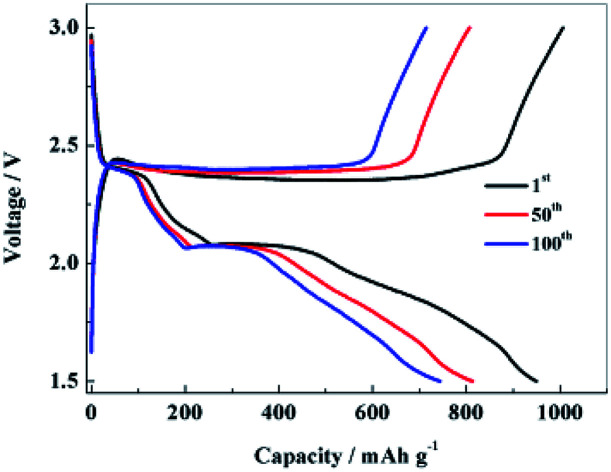

## 1. Introduction

Lithium–sulfur (Li–S) batteries are highly promising next-generation power sources because of their high specific capacity, high energy density, low cost, environment friendliness, and abundance. However, the application of Li–S batteries has several drawbacks, including the nonconductivity of sulfur, large volume change during cycling, and the shuttle effect of soluble lithium polysulfides. These problems lead to the deterioration of battery-cycle performance, which is a major concern of researchers. Accordingly, numerous related articles have been reported.^1–12^ Various methods have been adopted to improve the low electronic and ionic conductivities and poor cycle performance of sulfur. Initially, the researchers found that using porous carbon as a carrier of sulfur can significantly improve the performance of lithium–sulfur batteries. Many innovative contributions have been reported.^13,14^ The pores produced with the carbons examined appear to be small and this tends to limit the efficacy of these materials for Li–S materials either due to transport^15^ or limited sulfur loading.^16^ The surface functionalization of carbon materials has been also attempted to inhibit polysulfide dissolution. The pore structure of various carbonaceous materials (*e.g.*, graphene, carbon nanotubes, carbon fibers, and porous carbon) has been controlled to synthesize materials with high specific surface area and wide pore-size distribution. Thereby, the contact between sulfur and conductive substrate is enhanced, and the dissolution of polysulfides into the electrolyte is inhibited. Doping with heteroatoms (*e.g.*, nitrogen,^17–24^ sulfur,^25^ phosphorus,^26^ boron,^27^ and platinum^28^) can improve the conductivity and electrochemical activity of carbon materials. Additionally, there has been significant efforts to improve upon these materials through heteroatom doping of the porous carbons with nitrogen and sulfur doped materials reported extensively for both Li–S^29–31^ and Na–S^32,33^ batteries. Nitrogen doping can assist mesoporous carbon in inhibiting the shuttle effect and enhancing the interaction among nitrogen-doped-site functional groups and polysulfides. Thus, the electrochemical performance of Li–S batteries can also be improved. Meanwhile, sulfur doping can enhance the affinity between polysulfide and carbon substrate and thus the cycle performance of batteries. Even some nitrogen–sulfur dual-doped studies have been reported.^34–38^ However, in most cases, structural regulation and functionalization are two separate processes that result in complex preparation. Uneven doping and low doping levels also result from functionalization after the formation of complex pore structures. Thus, whether the one-step and one dual-doped carbon matrix can improve the performance of lithium–sulfur batteries remains unclear. A novel carbonaceous material with high specific surface area, wide pore-size distribution structure, and a special surface chemistry needs to be designed. Such structure should be able to carry a large amount of sulfur and provide a fast lithium-ion or electron transport channel. Such structure should also be able to enhance the interaction of sulfur/polysulfide with the carbon matrix to improve the rate performance and cycle stability of Li–S batteries.

In the present work, we introduced nitrogen and sulfur simultaneously by using one reagent to obtain heteroatom-doped porous carbon spheres. We used starch as the carbon source and l-methionine (C_5_H_11_O_2_NS, CH_3_–S–CH_2_–CH_2_–CH(NH_2_)–COOH) as the nitrogen and sulfur source. After a simple hydrothermal treatment, KOH activation, and hot-melt diffusion, the nitrogen–sulfur dual-doped porous carbon sphere/sulfur composite (PCS-NS/S) sample was obtained. It possessed wide pore-size distribution ranging from micropores to mesopores, held enough sulfur, withstood the volume expansion during charge–discharge process, and provided fast ion/electron transport channels. Given the wide distribution of the pore structure and surface functionalization of the PCS-NS/S sample, it exhibited high discharge capacity, excellent rate performance, and superior cycle stability.

## 2. Experimental

### 2.1 Preparation of PCS-NS/S

Soluble starch (carbon source) was dissolved in deionized water to form a homogeneous solution at first. l-methionine (nitrogen–sulfur source) was added into the mixture under stirring. The mass ratio of l-methionine and soluble starch is 1 : 5. Then after KOH activation, water washing and drying, the N,S dual-doped porous carbon spheres (PCS-NS) was synthesized. At last, PCS-NS was combined with sulfur after a hot melt-diffusion process, then the PCS-NS/S composites was obtained.

The specific preparation process is as follows: 7.2 g soluble starch (purchased from Tianjin Huadong Reagent Factory) and 1.44 g l-methionine (purchased from Sinopharm Chemical Reagent Co., Ltd.) were dissolved in 80 mL deionized water. Then the mixture was transferred into a sealed Teflon-lined autoclave and heated at 180 °C for 24 h. The product was collected by centrifugation and washed thoroughly with deionized water and absolute ethanol three times to remove the unreacted small molecular substances, and then dried in an oven at 60 °C for 24 hours. Then the sample was mixed with KOH uniformly and heat-treated in a nitrogen-protected tube furnace at 800 °C for 1 h. After washing with 1 M HCl and deionized water repeatedly and dried at 60 °C in air over night, the PCS-NS sample was achieved. Then 50 mg PCS-NS and 80 mg sublimed sulfur (purchased from Tianjin Fuchen Chemical Reagents Factory) were mixed uniformly, and heated at 155 °C for 12 h under a nitrogen atmosphere, at last the nitrogen–sulfur dual-doped porous carbon spheres/sulfur (PCS-NS/S) composites was obtained.

### 2.2 Characterizations of PCS-NS/S

X-ray diffraction (XRD) patterns were recorded on a Rigaku-Dmax 2500 diffractometer equipped with graphite mono-chromatized Cu Kα (*λ* = 0.154 nm) radiation at a scanning speed of 5° min^−1^ in the range of 10–80°. X-ray photoelectron spectroscopy (XPS) spectrums were performed on a PHI-5500 instruments spectrometer. Scanning electron microscopy (SEM) images were obtained using a Philips XL 30 and JEOL JSM-6700F microscopes. Transmission electron microscopy (TEM) images were produced with a Hitachi model H-8100 operating at 200 kV accelerating voltage. Nitrogen sorption–desorption measurements were performed on a Micromeritics ASAP 2020 analyzer. The specific surface area (SSA) was calculated using the Brunauer–Emmett–Teller (BET) method, and the pore size distribution (PSD) was calculated from the desorption branch based on the Barrett–Joyner–Halenda (BJH) equation. The sulfur content in the composites was measured in the temperature ranges from room temperature to 600 °C at a heating rate of 10 °C min^−1^ by using a TG/DTA 6300 in an N_2_ flow.

### 2.3 Fabrication of electrodes and battery assembly

PCS-NS/S (or S) served as the positive, whereas metal lithium acted as the negative electrode material in the batteries. The positive electrode slurry was prepared by mixing 80 wt% of the active materials, 10 wt% of the pvdf binder, and 10 wt% of the carbon black conductive agents in an *N*-methyl-pyrrolidone dispersant to form homogeneous slurry. The resulting slurry was uniformly coated on an aluminum foil current collector and dried at 120 °C for 5 h in a vacuum oven before assembling. The electrolyte was 1 M lithium bis(trifluoromethane) sulfonamide (LiTFSI) in 1,3-dioxolane/dimethoxymethane (DOL/DME, 1 : 1 by volume) with 0.1 M LiNO_3_ additive. The mass of electrode and volume of electrolyte was both important for practicality of the Li–S battery.^39^ In this paper, the mass of sulfur in positive electrode was controlled 2 mg approximately. The electrolyte volume of each coin cell was about 0.05 ml. The separator was Celgard 2400 polymer. All the cell assembly was carried out in an Ar-filled dry glove box (Mikrouna Co. Ltd.), in which the contents of both O_2_ and H_2_O were less than 0.5 ppm.

### 2.4 Electrochemical measurements

The galvanostatic charge–discharge tests of the coin cells were performed in the voltage range of 1.5–3 V. Electrochemical impedance spectra (EIS) and cyclic voltammetric (CV) tests were performed in a coin cell, which consisted of PCS-NS/S electrode as the working electrode and Li metal electrodes as both the counter and reference electrode. The potential of the working electrode (PCS-NS/S) was scanned between 1.5 and 3.0 V *vs.* Li.

## 3. Results and discussion

### 3.1 Characterization of PCS-NS/S

**Fig. 1:**
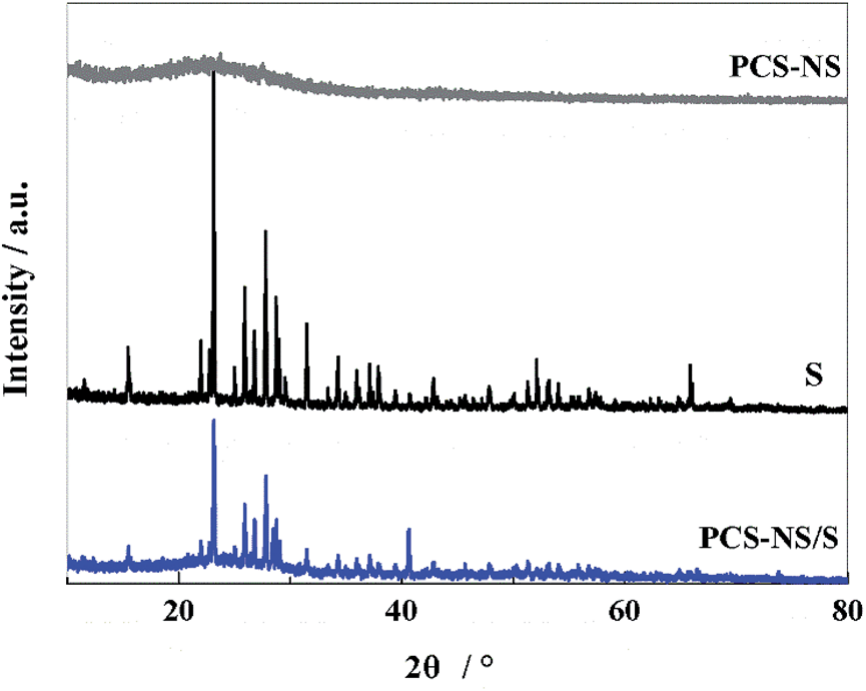
XRD patterns of PCS-NS/S, PCS-NS and S.

The crystal structure and phase composition of the samples were characterized by XRD tests. As shown in Fig. 1, PCS-NS displays a distinct wide and weak peak at around 24°, which corresponds to the graphite structure (002) plane of carbon, indicating that PCS-NS had an amorphous structure. After thermal combination with sulfur, PCS-NS/S composites showed a distinct sulfur diffraction peak, while the characteristic peak of carbon at 24° was maintained, indicating that sulfur had been combined with the PCS-NS substrate very well. The diffraction peak of sulfur should be caused by the sulfur outside of the carbon spheres.

**Fig. 2:**
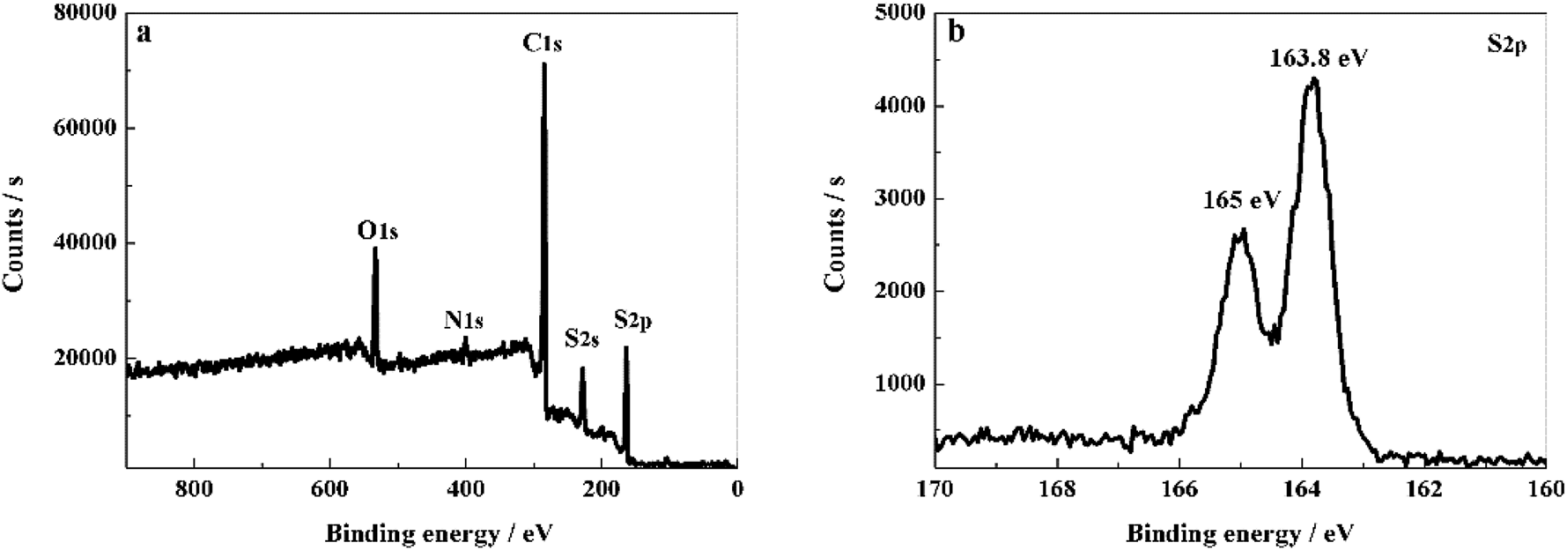
XPS survey of PCS-NS/S (a) and high resolution XPS spectra of S 2p (b).

XPS method was applied to determine the element species in PCS-NS/S composites and the interaction between the sulfur and the PCS-NS matrix. As shown in Fig. 2a, the XPS spectrum of PCS-NS/S composites behaved five main characteristic peaks at 533, 401, 285, 228, and 166 eV, corresponding to O 1s, N 1s, C 1s, S 2s, and S 2p peaks, respectively. Based on the high-resolution XPS spectra analysis of S 2p (Fig. 2b), two characteristic peaks located at around 163.8 and 165 eV can be seen, which correspond to the C–S–C and the C=S bond, respectively. Thus, after compounding with PCS-NS substrate, a strong interaction occurred between the carbon substrate and sulfur. This interaction has a strong limit effect on sulfur and polysulfide during the charge–discharge process and also effectively improved the electrochemical performance of batteries.

**Fig. 3:**
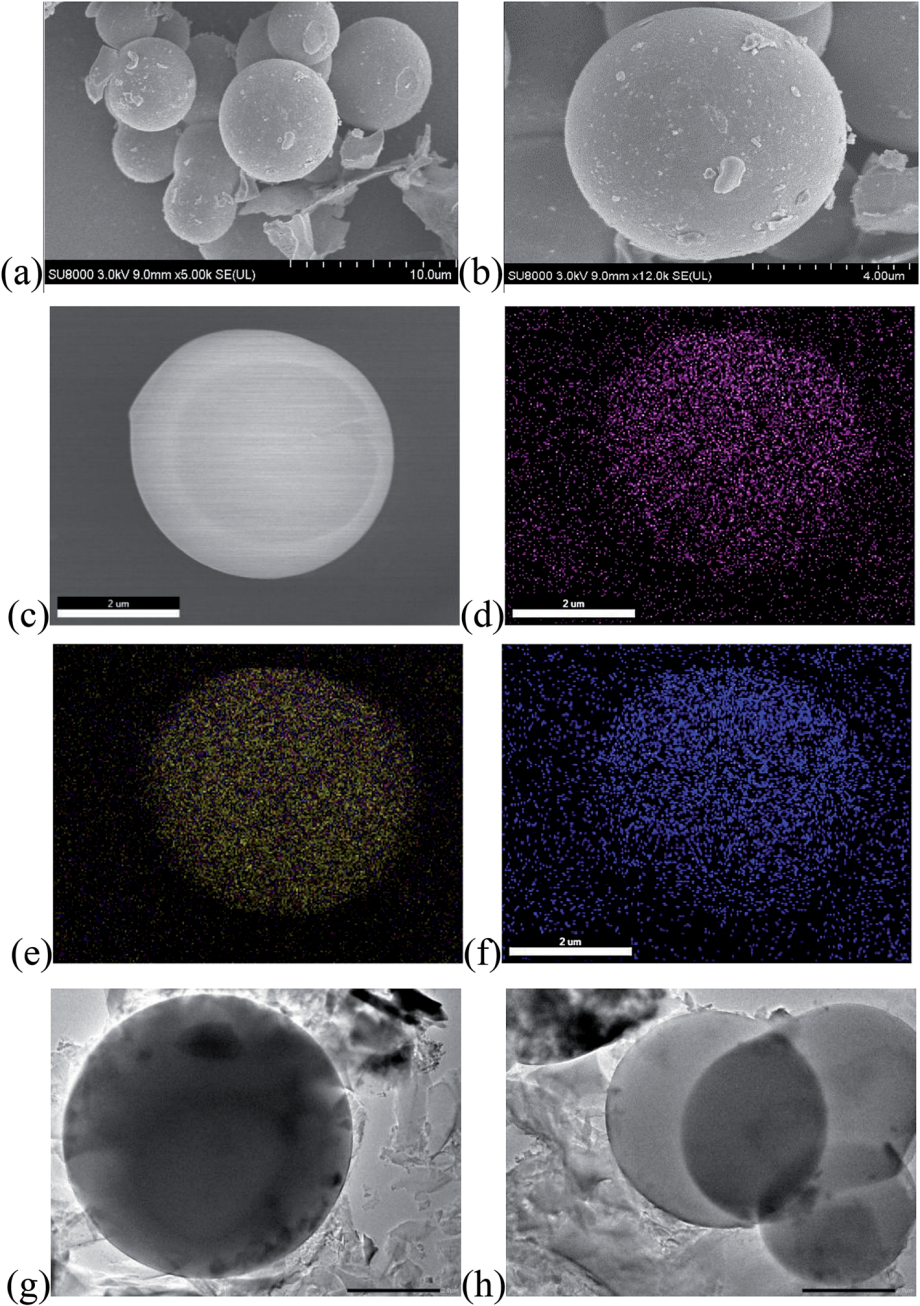
SEM images of PCS-NS/S (a–c) and corresponding elemental mappings of C (d), S (e), N (f) and TEM images of PCS-NS/S (g and h).

Morphological and structural analyses of PCS-NS/S composites were performed by using SEM and TEM tests. The results showed that the PCS-NS/S sample had a uniform spherical morphology with a diameter of approximately 3–5 μm. The elemental surface scan of PCS-NS/S (Fig. 3d and e) further shows that nitrogen and sulfur were uniformly distributed throughout the carbon matrix. Thus, l-methionine as the nitrogen–sulfur source can be well doped into the bulk phase of the carbon material.

**Fig. 4:**
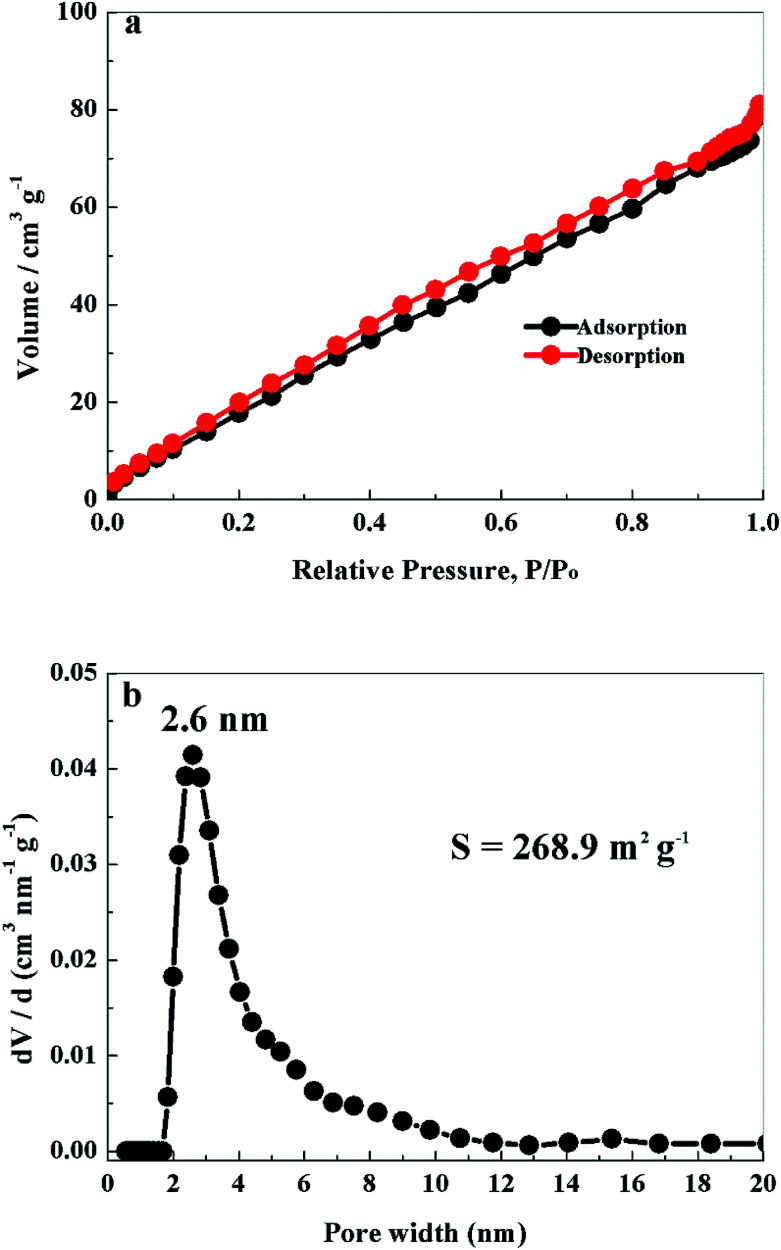
N_2_ adsorption–desorption isotherms (a) and the pore size distribution (b) of PCS-NS/S.

Nitrogen adsorption–desorption tests were performed to investigate the pore structure and specific surface area of PCS-NS/S composites. The equilibrium isotherm is shown in Fig. 4a, which is classified as an IUPAC type IV shape. As shown in Fig. 4b, the pore-size distribution of the PCS-NS/S sample was mainly distributed in 1.8–20 nm and was especially concentrated at 2.6 nm. The specific surface area of the PCS-NS/S composites was about 268.9 m^2^ g^−1^. The above results revealed that the PCS-NS/S composites had a wide pore-size distribution, ranging from micropores to mesopores and a large specific surface area. This microporous–mesoporous co-existing structure and the large specific surface area can facilitate electrolyte infiltration, inhibit polysulfide dissolution, and promote lithium-ion migration.

**Fig. 5:**
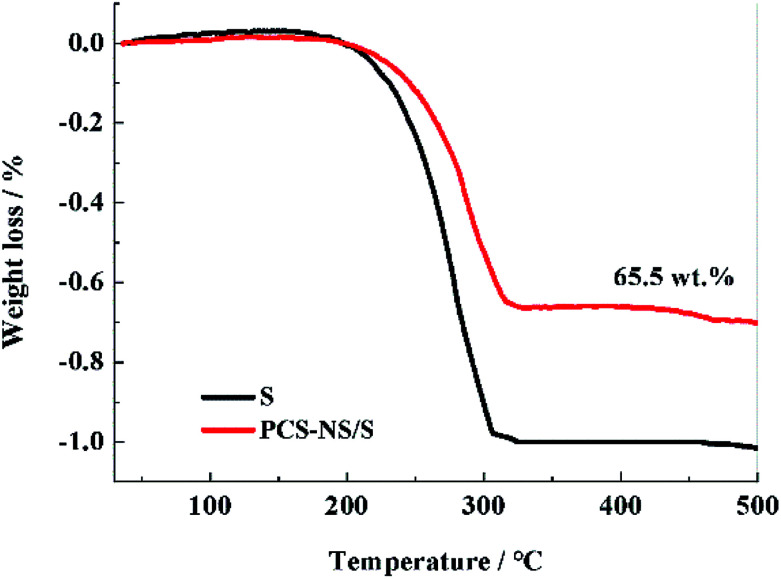
TG curves of PCS-NS/S and S samples.

Thermogravimetric tests were performed to determine the sulfur content in the PCS-NS/S composites. Fig. 5 shows the thermogravimetric curves of PCS-NS/S and sulfur. Based on calculations according to the thermal weight loss interval, the weight ratio of sulfur in PCS-NS/S composites was about 65.5%. The thermogravimetric curve placed two platforms between 160 and 400 °C. The first platform (low-temperature region) should correspond to the sulfur in the outer surface of the porous carbon, whereas the second platform (high-temperature region) corresponds to the sulfur in the pores of the porous carbon. The thermal stability of PCS-NS/S composites was superior to that of sulfur, owing to the capillary force of the pore structure of the PCS-NS matrix. This capillary force can limit the volatilization of sulfur obviously.

### 3.2 Electrochemical performance of PCS-NS/S

**Fig. 6:**
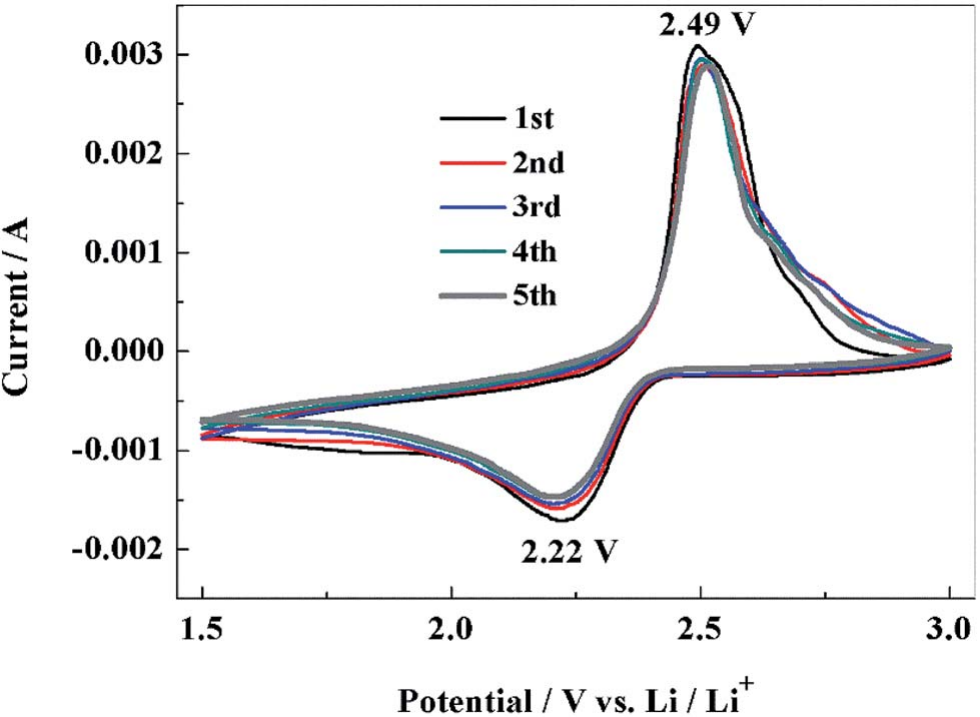
Cyclic voltammograms of PCS-NS/S electrode at 0.1 mV s^−1^ in the potential range of 1.5–3 V (*vs.* Li/Li^+^).

With PCS-NS/S as the positive electrode and lithium metal as the negative electrode, the lithium–sulfur batteries were assembled, and the electrochemical performance was investigated. Fig. 6 shows the CV response of the NS-PCS/S composite positive electrode recorded at a scan rate of 0.1 mV S^−1^. The initial discharge curve showed a significant reduction peak at 2.22 V, corresponding to the conversion of sulfur to high-valent polysulfide ions.^40^ In the reverse scanning, an oxidation peak appeared at 2.49 V, corresponding to the oxidation process and the formation of sulfur. The redox peak was narrow in shape, and the CV curves can be well overlapped, indicating that the PCS-NS/S positive electrode had good electrochemical reversibility and reaction kinetics. PCS-NS/S retained its good conductivity, which can inhibit the dissolution of polysulfide during charge–discharge process, exhibiting good electrochemical performance.

**Fig. 7:**
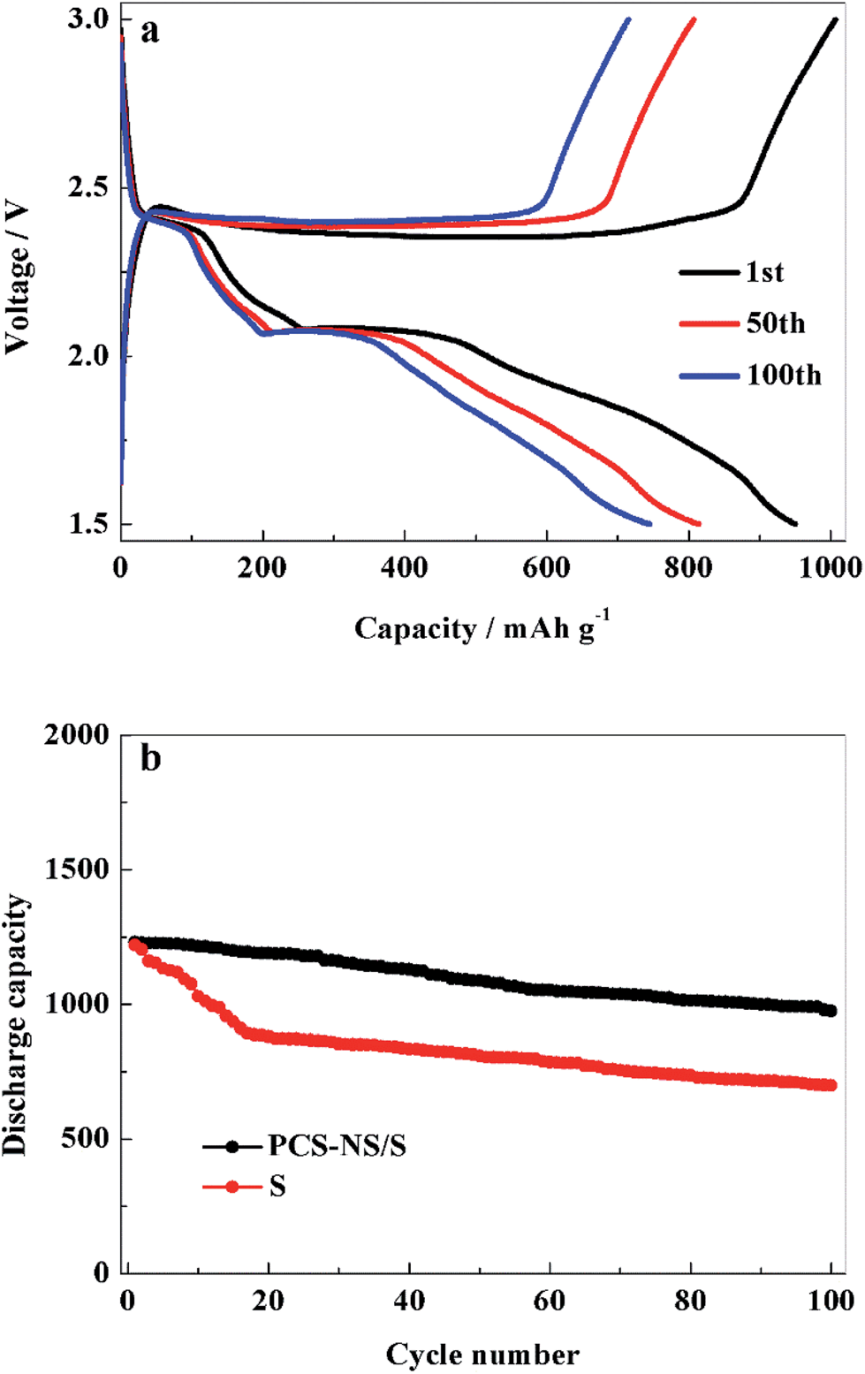
The charge–discharge curves (a) and cycling stability curves (b) of PCS-NS/S electrode between 1.5 and 3 V (*vs.* Li/Li^+^).

Galvanostatic charge–discharge tests of the batteries were performed to study the electrochemical performance of PCS-NS/S composites further. Fig. 7a shows the 1st, 50th, and 100th charge–discharge curves of the PCS-NS/S positive electrode at 0.3C. The first charge–discharge curve had an obvious discharge platform at about 2.2 V and a charge platform at around 2.5 V. This observation was consistent with the CV tests in Fig. 6. At a rate of 0.3C, the initial discharge capacity of the PCS-NS/S positive electrode was 950 mA h g^−1^, which is a good initial discharge capacity. After 100 cycles, the discharge capacity also remained at 745 mA h g^−1^, and the discharge curve kept a stable discharge platform, indicating that the PCS-NS/S composites had good cycle stability. Fig. 7b shows the cycle performance of PCS-NS/S at a rate of 0.1C. The initial discharge capacity of PCS-NS/S was 1230 mA h g^−1^, with the discharge capacity remaining at 974 mA h g^−1^ after 100 cycles, holding capacity retention of 79.2%. For comparison, the cycle performance curves of sulfur were also analyzed, and after 100 cycles, the discharge capacity was reduced from the initial 1221 mA h g^−1^ to 699 mA h g^−1^, with the capacity retention rate of only 57.2%. This finding indicates that PCS-NS/S had a superior electrochemical performance than sulfur, and it reduced the loss of active materials during charge–discharge process. Unfortunately, the capacity still behaved a certain degree of attenuation. Probably because the structure of this material was not perfect enough, which could not completely prevent the shuttle effect. It also can be seen that the cycle performance of the PCS-NS/S composite material was significantly better than that of the elemental sulfur only during the initial about 40 cycles. After 40 cycles, the fade rate of these two materials seemed exactly the same. The doped materials in this case appears to prevent the initial burst and loss of sulfur, but under longer cycling there is not a significant difference in the fade rate. It may be that the structure of the PCS-NS/S composite material suffered some damage during the cycle progress. So the shuttle effect was not well prevented beyond 40 cycles and the capacity was rapidly attenuated. But here, we have to admit that we could not explain the reason and mechanism very clearly now. We are willing to do some further research in the future.

**Fig. 8:**
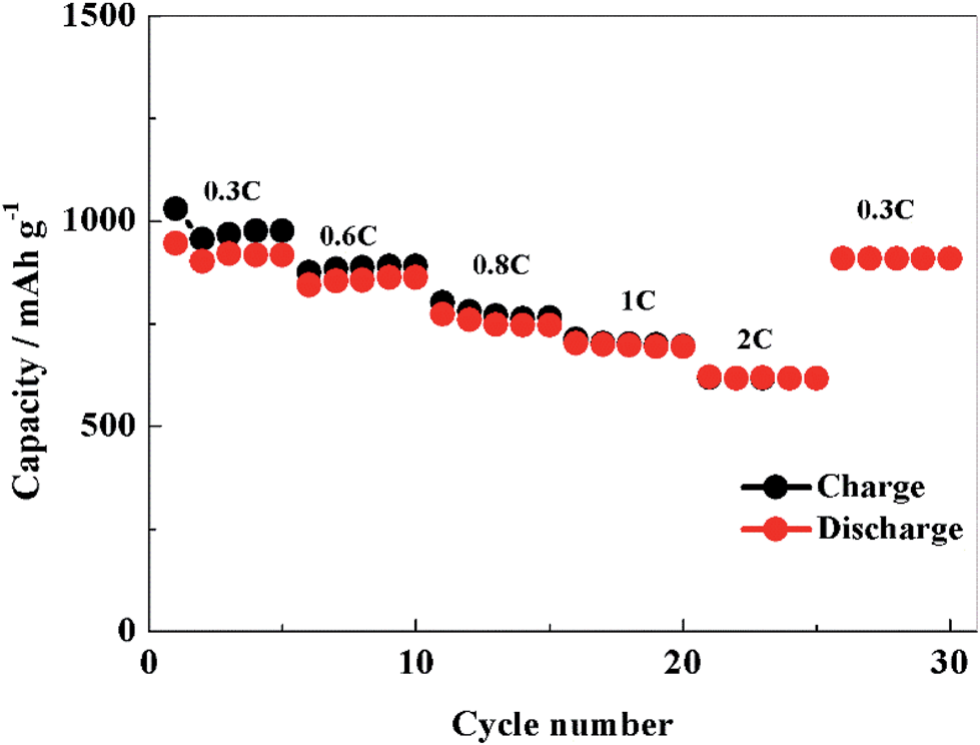
Rate performance of PCS-NS/S positive electrode in the voltage range of 1.5–3 V.

Fig. 8 shows the rate performance of PCS-NS/S composites, which is tested at room temperature at 0.3, 0.6, 0.8, 1 and 2C, respectively. The initial discharge capacity of PCS-NS/S was as high as 945 mA h g^−1^ at 0.3C. When the rate was raised to 2C, the discharge capacity was 620 mA h g^−1^. After continuous discharged at 0.6, 0.8, 1 and 2C, the discharge capacity can be still restored at 909 mA h g^−1^ when the rate returned to 0.3C. Obviously, this performance has a relatively high rate. Moreover, the PCS-NS/S composites possessed a good charge–discharge efficiency at the same time.

**Fig. 9:**
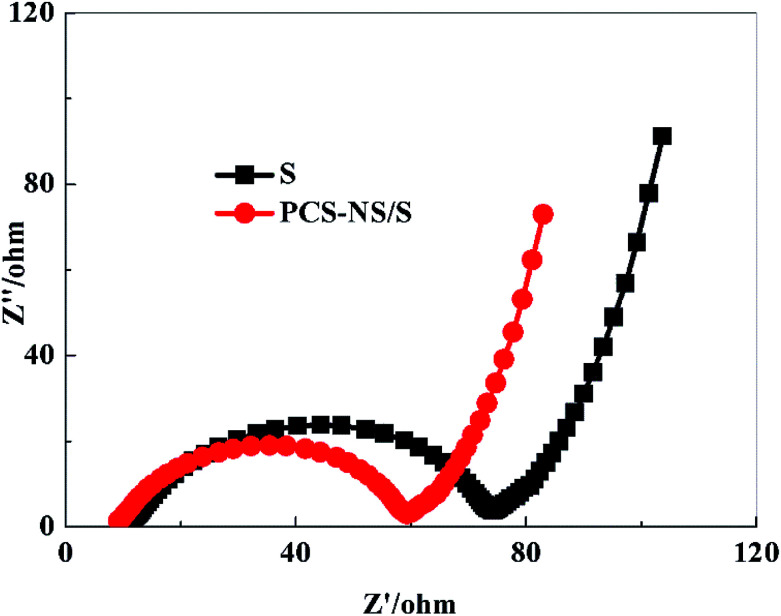
Electrochemical impedance spectra (EIS) of PCS-NS/S and sulfur

Fig. 9 is the EIS spectrum of the PCS-NS/S composites and sulfur positive electrode before charging–discharging with a frequency range from 100 kHz to 10 MHz. All the impedance spectra consisted of a depressed semicircle in the high-medium frequency region followed by a slanted line in the low frequency region. The semicircle corresponds to the charge transfer resistance (*R*_ct_) at the electrode/electrolyte interface, and the straight line in the low-frequency domain corresponds to a semi-infinite Warburg diffusion process.^41^ The ohmic impedance and charge transfer resistance of PCS-NS/S composites were both smaller than that of sulfur, indicating that the conductivity of PCS-NS/S composites was improved apparently.

## 4. Conclusions

PCS-NS/S composites were prepared through a facile one-step hydrothermal method by using starch as carbon source and l-methionine as nitrogen–sulfur source. The structure, morphology, pore structure, and sulfur content were investigated in detail. Physical properties indicated that PCS-NS/S has high specific surface area, wide pore-size distribution, and high sulfur loading. Electrochemical tests showed that doping nitrogen and sulfur can introduce numerous surface active sites in the porous carbon spheres, which can effectively inhibit the dissolution and shuttle effect. As a lithium–sulfur positive electrode material, PCS-NS/S composites exhibited high discharge capacity, high capacity retention, good rate performance, and superior conductivity.

## Conflicts of interest

There are no conflicts to declare.

## Supplementary Material
